# Network Analysis of Time Series: Novel Approaches to Network Neuroscience

**DOI:** 10.3389/fnins.2021.787068

**Published:** 2022-02-11

**Authors:** Thomas F. Varley, Olaf Sporns

**Affiliations:** ^1^Department of Psychological and Brain Sciences, Indiana University, Bloomington, IN, United States; ^2^School of Informatics, Computing, and Engineering, Indiana University, Bloomington, IN, United States

**Keywords:** network science, complex system, information theory, manifold learning, time series analysis, recurrence analysis, visibility graph, ordinal partition network

## Abstract

In the last two decades, there has been an explosion of interest in modeling the brain as a network, where nodes correspond variously to brain regions or neurons, and edges correspond to structural or statistical dependencies between them. This kind of network construction, which preserves spatial, or structural, information while collapsing across time, has become broadly known as “network neuroscience.” In this work, we provide an alternative application of network science to neural data: network-based analysis of non-linear time series and review applications of these methods to neural data. Instead of preserving spatial information and collapsing across time, network analysis of time series does the reverse: it collapses spatial information, instead preserving temporally extended dynamics, typically corresponding to evolution through some kind of phase/state-space. This allows researchers to infer a, possibly low-dimensional, “intrinsic manifold” from empirical brain data. We will discuss three methods of constructing networks from nonlinear time series, and how to interpret them in the context of neural data: recurrence networks, visibility networks, and ordinal partition networks. By capturing typically continuous, non-linear dynamics in the form of discrete networks, we show how techniques from network science, non-linear dynamics, and information theory can extract meaningful information distinct from what is normally accessible in standard network neuroscience approaches.

## 1. Introduction

Over the course of the last decade “network neuroscience” has emerged as a rapidly expanding research paradigm in computational and cognitive neuroscience (Sporns, 2010; Fonito et al., 2016). A core breakthrough has been the development and refinement of the idea of the “connectome” (Sporns et al., 2005), which generally refers to a network model that encodes all pairwise connections between elements of the brain (or, if we're feeling particularly ambitious, the whole nervous system). In a connectomics model, the nodes of the network are naturally understood as neurons, brain regions, or other components of the nervous system and the edges are physical connections (e.g., white matter tracts) or statistical dependencies (functional or effective connectivity). The resulting network can be weighted, unweighted, directed, or undirected and methods developed in graph theory and network science provide insights into the systematic organization of the brain, the underlying biology, and alterations seen in disease (Sporns, 2010; Fonito et al., 2016).

Functional connectivity networks have become a particular object of interest among network neuroscientists (van den Heuvel and Hulshoff Pol, 2010; Smith et al., 2011). The appeal of functional connectivity networks is obvious: by providing a map of how different brain regions couple, it becomes possible to decompose the whole system into sets of interacting elements, or sub-networks (Yeo et al., 2011), which can be analyzed independently. There is a price to be paid for constructing functional connectivity networks, however: in its raw form, the data that comes out of many neuroimaging modalities (fMRI, M/EEG, single-neuron recordings, etc.) contains both spatial information (in the sense that every recorded element has its own unique time series) and temporal information, recorded as a multidimensional time series. Functional connectivity analysis can be thought of as “collapsing” across the temporal dimension, while preserving the spatial distinction between elements. The individual elements are preserved as distinct nodes, while much of the rich temporal dynamics are lost and replaced with a vector-valued summary statistic indicating some sort of coupling between elements (typically the expected value of some statistic: e.g., Pearson correlation, transfer entropy, etc.) (Friston, 1994).

This collapsing often throws out important dynamical information, which can reveal how the system evolves through time. This is a core area of research in modern computational neuroscience—understanding the low-dimensional structure of neural dynamics (described as dynamics on a “manifold”) (Woodman and Jirsa, 2013). While the nervous system can have functionally infinite degrees of freedom (corresponding to the number of neurons, or voxels in a dataset), correlations between elements can constrain the evolution of the whole system to a subspace of the possible global state-space (the manifold). These low-dimensional neural manifolds have been found at every level of neural function: ensembles of spiking neurons (Gallego et al., 2017, 2018, 2020; Chaudhuri et al., 2019), electrophysiological recordings (Kuo et al., 2018; Varley et al., 2021b) whole-brain fMRI studies (Shine et al., 2018, 2019a,b), and have been conjectured to relate to differences associated with mental illness and psychopathology (Carhart-Harris and Friston, 2019; McIntosh and Jirsa, 2019). Conceptually, the idea of a neural manifold can be understood as a “space”, every point of which corresponds to a possible “state” a system can be in. The evolution of the system through time can be understood as a flow, or path, traversing the state-space landscape. Modeling these manifolds, and the particular path the system traverses over them, requires understanding how the instantaneous states at different moments in time (typically individual frames or time points of a multivariate time series) relate to each other and requires time-resolved analysis of the whole system, rather than global measures of interaction.

Typically, manifolds are modeled as continuous surfaces, using differential equation models (Huys et al., 2014), or dimensionality-reduction algorithms like PCA (Shine et al., 2018) or diffusion-map embedding (Margulies et al., 2016). While continuous embeddings provide insights and don't automatically require coarse-graining, they come with inevitable practical limitations when compared to discrete models, and in fact, many “continuous” manifold-embeddings use discrete nearest-neighbor networks to approximate distances in high-dimensional spaces [e.g., tSNE (Hinton and Roweis, 2003), UMAP (McInnes et al., 2018), or KSG information estimators (Kraskov et al., 2004)].

Historically, there has been considerable interest in creating discrete state-space and state transition models of continuous brain dynamics. Popular approaches may include unsupervised clustering algorithms (e.g., *k*-means clustering) or inferring hidden Markov models. While both have been informative (discussed blow), a common limitation of these measures is typically small number of “states” (often <10), which is likely not enough to fully characterize a manifold. Combined sliding-windows functional connectivity analysis and *k*-means clustering can reveal differences in the state transition structure of the brain under different conditions (Li et al., 2019; Lord et al., 2019; Schumacher et al., 2019; Singleton et al., 2021), however a state-space model of 5–10 unique “states” may be too impoverished to extract detailed information about the structure of the intrinsic neural manifold. Hidden Markov models have been similarly applied to multiple neuroimaging modalities, including spiking networks in rodents (Jones et al., 2007), MEG data from humans (Baker et al., 2014), and fMRI data from humans (Eavani et al., 2013; Chen et al., 2016). As with *k*-means based state-detection pipelines, the number of states that can be discerned is typically less than 15.

In this paper, we describe a suite of alternative methods of inferring discrete manifolds (in the form of networks) from neural data. In contrast to *k*-means and HMM-based models, these techniques are finely time-resolved and can allow for the distinction of hundreds of unique micro-states. Collectively referred to as “network analysis of time series” (Lacasa et al., 2008, 2015; Donner et al., 2010; Small, 2013; McCullough et al., 2015; Zou et al., 2019), these methods aim to provide a best-of-both-worlds approach: allowing researchers to leverage the considerable power of graph theory and network science to understand neural manifolds without reducing the number of states to the same extent that more well-known standard algorithms do. We suggest that these approaches constitute a complementary branch of network neuroscience based on analyzing manifold networks rather than functional or structural connectivity networks. This kind of network-based analysis can be thought of as “orthogonal” to traditional, connectome-based analyses. Instead of preserving spatially distinct elements as nodes and collapsing temporal dynamics into edges, in network analysis of time series, the whole system (or time series) at a given instant is collapsed into a single state vector, and the edges (which can be directed, undirected, weighted, or unweighted) correspond to movement through a state-space. Local temporal dynamics become encoded in the local graph neighborhood of a single node, allowing every moment to be assessed independently, while global properties of the whole dataset are encoded in the global structure and topology of the network.

Network-based analysis of time series is a comparatively novel field and has only begun gaining momentum in the last few years. Much of its history can be traced back to analysis of non-linear signals, specifically (although not exclusively) techniques for attractor reconstruction (Takens, 1981) and recurrence analysis. The basic motivation for attractor-reconstruction is to try and infer the overall “state-space landscape”: the set of states that the system under study can adopt, how it is likely to evolve through time (corresponding to a trajectory through the state-space), and what it's long-term behavior might look like. A particular set of states that the system gets “stuck” in long-term is called the “attractor.” The canonical example of non-linear attractor analysis is the discovery of the Lorenz Attractor, and by extension, dynamical chaos, in an atmospheric model (Gleick, 2011). Since then, dynamical systems models have been applied in a variety of fields, including neuroscience to understand the different dynamics driving different cognitive states and behaviors (Chaudhuri et al., 2019; Shine et al., 2019a). In classical attractor reconstruction analysis, a continuous, non-linear signal is “embedded” in an *n*-dimensional space using a time-delay embedding (described in detail below), resulting in a point-cloud, where every moment in the initial time series corresponds to a vector in *n*-dimensional space. Analyzing the structure of this point-cloud, such as its topology, correlation dimension, or chaosticity, can provide insights into the generating processes that gave rise to that signal (for an overview of these concepts and others from chaos theory, see Strogatz, 2018). Network analysis of time series follows a similar pipeline. Every moment in the time series is mapped to a node in a network, and edges are assigned to nodes according to some criteria (the specifics of which vary with the construction algorithm). In this paper, we explore three ways of constructing a network from time series:

1. *Recurrence Networks*: Networks which encode the tendency of the system to return to, or dwell in, particular subspaces (or macro-states) as it evolves over a continuous manifold. See Section 2
2. *Visibility Networks* Networks which encode the structure of extreme and common events throughout the duration of the time series. See Section 3
3. *Ordinal Partition Networks*: Networks which encode probabilistic state transition dynamics through a space of discrete system “states” in a manner similar to a finite state machine. See Section 4

As with continuous attractor-embeddings, analysis of the topology of the resulting networks can provide insights into dynamics of the original time series. One significant benefit of this method, however, is that a network is a discrete, rather than continuous manifold, which allows us to leverage the considerable work that has been done at the intersection of graph theory and information theory (Cover and Thomas, 2012; Klein and Hoel, 2020; Rosas et al., 2020) to understand information dynamics in brain data in ways that cannot be as easily done when operating on a continuous signal.

In this paper, we follow the outline provided by Zou et al. (2019), who lay out a mathematically rigorous and extremely comprehensive introduction to multiple methods of constructing networks from time series data. As we are writing for an audience of neuroscientists, we will focus less on the mathematical details, and instead on the significance of these methods in the explicit context of neural data. We encourage the interested reader to refer either to Zou et al. (2019) or any of the cited primary source papers for further details. In addition to providing the technical details of how these networks can be constructed and analyzed, we have taken pains to propose intuitive interpretations of the various metrics proposed here, particularly as they might relate to neural data. As network neuroscience has developed, a critique of the field has been that the development of mathematical techniques has often outpaced scientific interpretation, resulting in studies where researchers apply analyses or calculate metrics without a clear understanding of what those metrics mean in the context of the data being sampled (De Vico Fallani et al., 2014; Hallquist and Hillary, 2018). Graph metrics are mathematically well defined, but the biological interpretation is often not obvious, particularly when operating on structures such as fMRI connectivity networks, which are themselves statistical abstractions created from an indirect measure used to infer neural activity. By providing intuitive interpretations of the various network measures proposed here, we hope that this new branch of applied mathematics can be made accessible to neuroscientists, biologists, and cognitive scientists who can use it to answer outstanding about the brain and mind.

## 2. Recurrence Networks

Recurrence Networks (RNs) were the first proposed method of constructing networks from embedded time series (Donner et al., 2010), and can be built using both univariate and multivariate time series. The intuition behind a RN is that the evolution of a complex system through time can be thought of as a trajectory over some kind of manifold (for a 128-channel EEG array, each state would be a point in a 128-dimensional space), and that, over time, the system will return to subsets of the state-space that it has already visited (Strogatz, 2018). If we consider each moment in the time series as a node in the network, then we can then draw an undirected edge between two nodes if the “distance” between the two points on the manifold (typically formalized with a metric such as an L_*p*_ norm) is less than some arbitrary value of ϵ. RNs are isomorphic to the notion of a recurrence plot (Eckmann et al., 1987), as the recurrence plot is taken as the adjacency matrix defining a RN. The RN is very powerful in that, unlike most other methods for analysis of time series, there is no requirement that the underlying data be regularly sampled, since there is no temporal information encoded directly in the network. Missing bins and irregular sampling rates are nonissues since every node is treated as a random sample from the underlying manifold: RNs have been used to analyse irregularly-sampled paleoclimate data (Donges et al., 2011a,b). This could potentially make recurrence networks extremely useful for analyzing behavioral data (particularly longitudinal studies) which is often irregularly sampled [a similar algorithm—Recurrence Quantification Analysis has been used in longitudinal behavioral data (Brick et al., 2017; Danvers et al., 2020)].

An RN can be thought of as one way of approximating an attractor driving a system under observation. The most basic method of constructing an RN assigned a weight of 1 to all edges with distance less than ϵ, regardless of the actual metric. The result is one of the simplest kinds of networks: an unweighted, undirected network, which will admit almost all of the standard algorithms in network science. Alternately, we can preserve “spatial” information by assigning the actual metric between two points to the weight of the edge connecting them, resulting in an undirected, weighted network. The weighted network contains significantly more information than the unweighted network and can be useful when doing a spatial embedding where relative distances are meaningful. The weighted network also has the advantage that all edges are strictly positive (in contrast to a Pearson correlated-based functional connectivity network), removing the uncertainty around interpreting negative weights on networks.

One appeal of recurrence networks is that they encode many features of interest in classical recurrence analysis in new ways, as well as providing new analyses based on the global structure of the network. For an example, consider the sample entropy, which approximates the entropy rate of a continuous signal (Richman and Moorman, 2000; Lake, 2011). Given an embedding dimension *m* and a threshold ϵ, the sample entropy of a time series is:


$$S(X_{t})=-log(\frac{A}{B})$$

Where *A* is the number of recurrences when the embedding dimension is *m*+1 and *B* is the number of recurrences when the embedding dimension is just *m*. In the context of a recurrence network, *A* and *B* become the number of edges in the respective networks and the sample entropy is the log-ratio of the two network densities. As an approximation of entropy rate (Lake, 2011), sample entropy is typically used as a measure of the “complexity” of a time series and multiple graph measures could be used to capture additional dimensions of “complexity”, such as the degree distribution, global efficiency, or clustering coefficient. While each of these new versions of sample entropy will have their own interpretations and mathematical properties (which we will not explore here), this example highlights how casting a recurrence plot as an RN can help facilitate new and potentially insightful analyses of dynamical processes.

### 2.1. Constructing a Recurrence Network

Recurrence networks can be easily constructed from multidimensional time series data, and most neural data comes in this form (e.g., BOLD signals from multiple parcels, multiple neurons recorded using an multi-electrode array, etc.). Recurrence networks can be constructed from univariate time series, in which case the first step is to perform a time-delay embedding on the series, using some appropriately selected embedding dimension *d* and lag τ (see Section 4.1)

Given some multivariate time series with *n* dimensions:


$$\begin{array}{l} {\text{X}}_{t}={\left(X_{1},X_{2},...,X_{t}\right)}_{|X_{i}\in {\mathbb{R}}^{n}}, \end{array}$$

Where each element *X*_*i*_ corresponds to an *n*-dimensional vector, we can teat each vector *X*_*i*_ as a point embedded in *n*-dimensional space. We then define a *t* × *t* matrix *M* and populate it such that:


$$\begin{array}{l} M_{ij}=\Theta \left(\epsilon -||X_{i},X_{j}||\right) \end{array}$$

**Figure 1:**
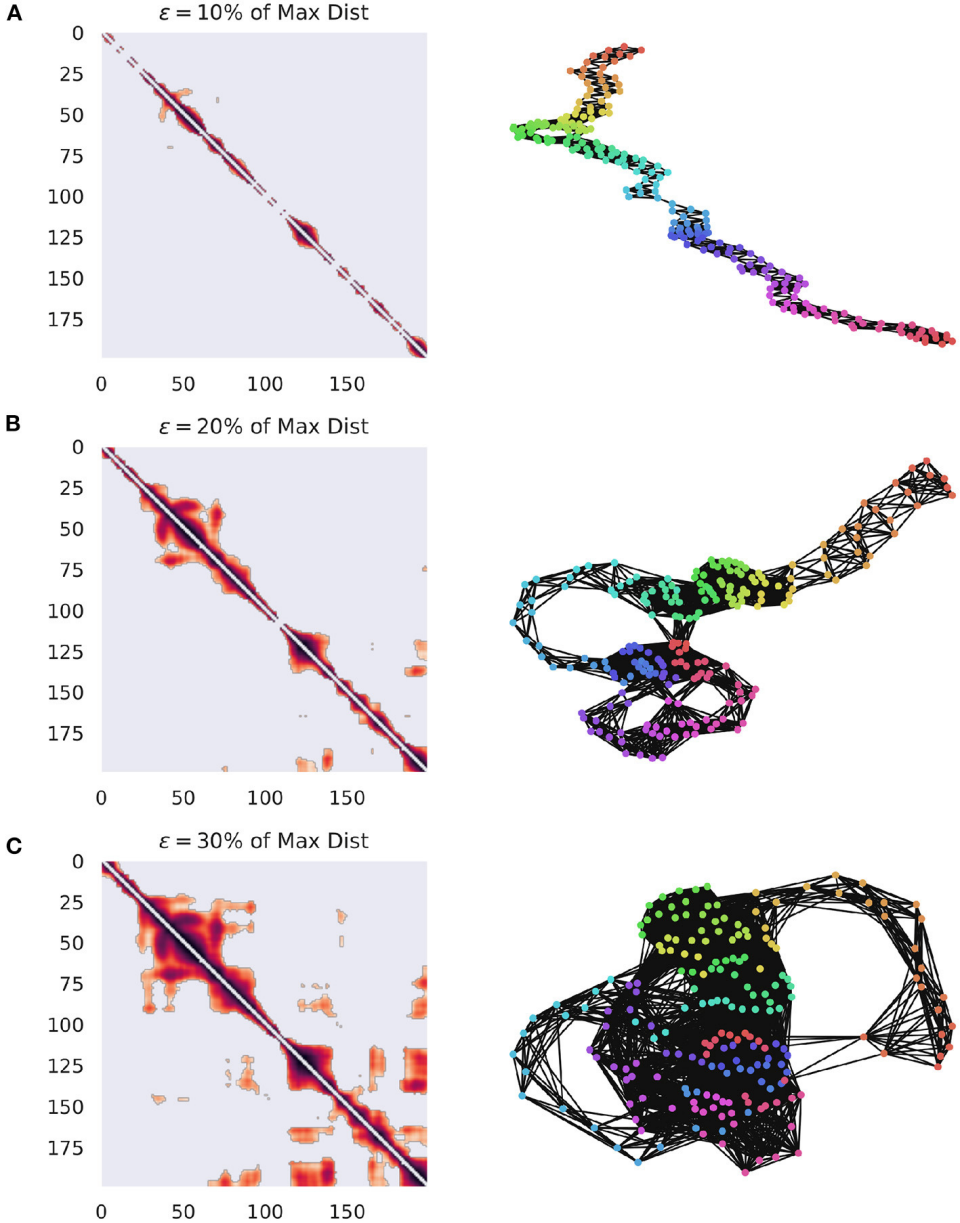
Visualization of how **G**_**R**_ changes when the value of ϵ is varied. The networks are constructed from ECoG data from the Neurotycho database and the distance function is the cosine distance (Nagasaka et al., 2011). **(A)** The weighted adjacency matrix and the associated RN thresholded at 10% of the maximal distance in the point cloud. It is clear that this is too low of a threshold, since every point is only similar to it's immediate past and future, creating a path graph. **(B)** The same network, this time thresholded at 20% of the maximum distance. Note that clear cyclic structures, indicating recurrences have started to appear, suggesting that the system is returning to particular regions of phase-space at distinct points in time. **(C)** The same network, this time thresholded at 30% of the maximum distance. This one captures even more meaningful recurrences, although at the cost of a much denser network.

Where Θ() is the Heaviside function and || || corresponds to some suitable distance measure. *M* can then be understood as the adjacency matrix for some binary, undirected network *G*_*R*_, whose sparseness varies inversely with ϵ. Other methods of matrix construction are possible, for instance we could define *M*_*ij*_ = *d*(*X*_*i*_, *X*_*j*_), for some distance metric *d*() which provides a fully-connected distance matrix for all pairs of points. This dense matrix could be thresholded at some arbitrary percentile or value of ϵ, thus producing a sparse network that preserves distance information. The specific value of ϵ is also relevant (Marwan et al., 2007; Marwan and Webber, 2015) as the network becomes fully dense as ϵ gets large (for a visualization of the effect of ϵ on network construction, see Figure 1). Furthermore, as ϵ grows larger than the mean distance between points, edges are likely to form between subsequent points, as opposed to temporally distinct recurrences. Several different heuristics for selecting the threshold have been suggested, usually focused on enforcing sparse networks, such as a few percent of the maximum distance (Mindlin and Gilmore, 1992; Zbilut and Webber, 1992; Koebbe et al., 1994), or to force a particular density of recurrences (edges in the network) (Zbilut et al., 2002). Thiel et al. (2002) argues that it is important to consider the effect of experimental noise on the structure of data, and that, given noise with a standard deviation of σ, ϵ should be selected such that ϵ>5σ, although this requires having a good estimate of the noise distribution of the data, which can be difficult to achieve. Numerical explorations of the effects of adding white and colored noise to the Lorenz System on the resulting RN have shown that noise can compromise the specific recurrence structure, however the ability of the network to preserve the overall structure of the attractor is remarkably robust (Jacob et al., 2016).

Other possible landmarks that leverage network analysis might include: the minimum value for which the network is fully connected, the value which maximizes the variance in the degree distribution (McCullough et al., 2015), or the value that optimizes the community structure of the network (for a discussion of community detection in RNs, see Section 2.2.1).

#### 2.1.1. Choosing a Distance Metric

The question of exactly which distance function to use is a non-trivial one. In low-dimensional systems, Euclidean distance can be used for an intuitive interpretation, however as dimensions grow, many distance metrics (Manhattan, Euclidean, Chebyschev, etc.) become increasingly uninformative (this is known as the curse of high dimensionality, Aggarwal et al., 2001). While there is no “right” answer for every context, the cosine distance is one appealing alternative to Lp-metrics as it does not suffer from the curse of dimensionality. For very high dimensional data, the Pearson correlation can serve as a distance metric as well.

If one is not concerned about losing regionally-specific information in the embedded vector (where each element corresponds to the instantaneous activation at the associated point in the brain or recording array), dimensionality reduction techniques can reduce the effects of the “curse of dimensionality.” If the dynamic activity in the brain is truly represented by a low-dimensional manifold, such an initial dimensionality reduction shouldn't compromise the integrity of the resulting network attractor (again, at the cost of loosing topographic information), although this remains an area requiring further exploration.

### 2.2. Analyzing a Recurrence Network

Many of the basic graph theoretic measures applied to RNs were explored in Donner et al. (2010) (which focuses on RNs constructed from time-delay embeddings of a univariate time series—for an explanation of such embeddings, see Section 4.1), and have subsequently been elaborated upon. The most basic measure that can be extracted from an RN is the number of edges (*network density*), which quantifies the ϵ-recurrence of the system under study. The degree (or normalized degree-density) of a node provides information about the local density of that region of phase-space: a high degree indicates the a dense region of phase space (i.e., the system visits this region a lot or spends a lot of time there), while a low, or zero degree suggests that the system was visiting a rare or unlikely configuration. Interestingly, Donner et al. (2010) and Zou et al. (2019) offer an argument that the local and global clustering coefficient provides information about the dimensionality of the underlying system: periods of time characterized by high clustering, where many points are all mutually ϵ-close are associated with an alignment of the state-vectors that suggests low-dimensional dynamics. The betweenness centrality of a node is thought to encode the local fragmentation of the system (Donner et al., 2010): as Zou et al. (2019) describes, if the attractor is characterized by several dense regions of phase space, connected by a small number of intermediate points, those points “bundle” shortest paths into a single walk characterized by a high degree centrality. Those nodes with high betweenness centrality indicate states that might serve as “bridge states” between two distinct, dense regimes. Finally, if the data is sampled regularly, the distance between two subsequent samples (*X*_*i*_(*t*) and *X*_*i*_(*t*+1)) is proportional to a measure of “velocity”, or how fast the system is evolving through the state space at a given instant. Previous analysis has found that the distributions of velocities reflects gross changes in brain states (Varley et al., 2021b).

In addition to the network-based measures, all the standard techniques from basic recurrence quantification analysis (RQA) can also be applied. For detailed reviews of these methods, see (Marwan et al., 2007; Marwan and Webber, 2015).

#### 2.2.1. Community Detection

One key, but rarely explored, application of recurrence networks is as a measure of where the state-space is “dense” and where it is “sparse.” As an analogy, consider a dataset that consists of random samples from a unimodal, bivariate Gaussian distribution. Regions of the distribution where the probability density is high (the peak of the “hill”) will see a higher density of samples, while the tails will not and the relative probability maxima can be determined based on the where the points are densest. This is the logic that underlies many K-nearest neighbors-based techniques for non-parametric probability estimation [e.g., the well-known KSG Nonparametric Mutual Information estimator (Kraskov et al., 2004)].

**Figure 2:**
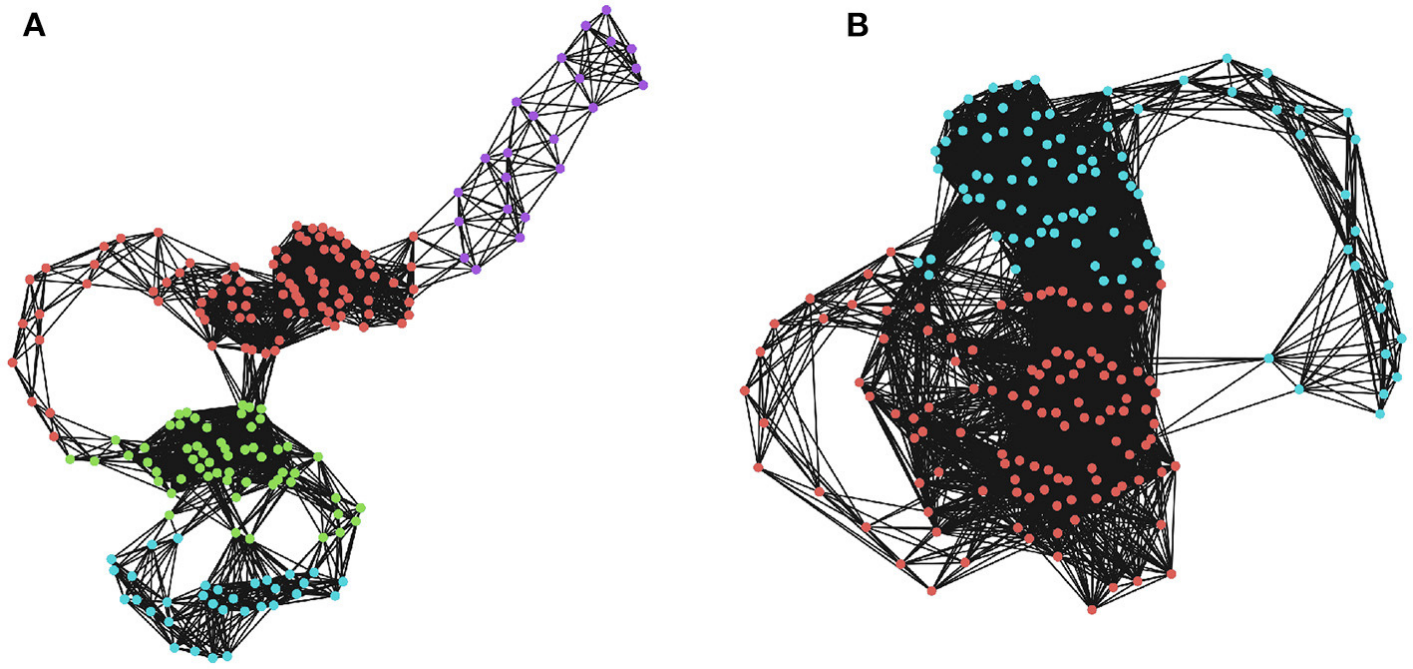
Community detection in recurrence networks. The effects of ϵ on the higher-order community structure of the RNs. Communities were determined using a greedy modularity maximization function in NetworkX (Hagberg et al., 2008). **(A)** The community structure when ϵ is 20% of the maximum distance. Here, there are four distinct communities corresponding to temporally distinct “regions” of the phase space that the system visits in sequence. **(B)** The community structure when ϵ is 30% of the maximum distance, which only returns two communities that are not as restricted in time. Depending on the threshold, two moments can be lumped into the same, or different macro-states.

A similar logic applies to higher-dimensional manifolds, although we should note that in a complex, non-linear time series, we cannot necessarily assume that each point is independently sampled from the manifold. Regions of the state-space that the system frequently returns to (or slows down upon visiting) are characterized by a large number of closely-spaced samples, which will become high-dimensional simplicial complexes, even for comparatively small values of ϵ. A community-detection algorithm could be used to find “macro-states”: those regions of the state-space that the system revisits or gets “stuck” in, and provide a more principled coarse-graining than selecting an arbitrary *k* for a *k*-means algorithm. The optimal community-detection algorithm to use on an RN remains uncertain and is an area worthy of future investigation, although standard techniques promise a useful starting point (for a visualization of community assignments for RNs of different densities, see Figure 2).

#### 2.2.2. Relationship to Topological Data Analysis

A key feature of the RN, in contrast to other methods described later, is that, under certain conditions, the recurrence network is able to preserve the topology of the underlying, continuous attractors (Donner et al., 2011), and therefore points to a natural association between RNs and an emerging field of topological data analysis [TDA; for an accessible general introduction, see (Chazal and Michel, 2017), for a discussion of TDA's relevance to modern neuroscience, see (Sizemore et al., 2018)]. TDA is used to assess the presence (or absence) of higher-order structure in large datasets, where every observation is treated as a point embedded in a high-dimensional space. In the case of neural data, this may be the distribution of instantaneous activity across a recording array (Varley et al., 2021b) or a point cloud constructed by embedding a single time series (Ouyang et al., 2008). The presence of densities and sparse regions (such as cycles, cavities, and higher-dimensional analogues of such voids) can provide insights into the structure of data and, in the context of embedded time series, the generating dynamics (Perea, 2018). The relationship between TDA and network analysis of time series is highlighted by recent work that takes a *precision dynamics* approach to understanding global state-transition dynamics in the brain (Saggar et al., 2021): by casting on going brain dynamics as a network of states, the authors found that certain states have hub-like characteristics that enable ongoing dynamics. Similar results are reported in Saggar et al. (2018) who used the Mapper algorithm (Singh et al., 2007) to construct a network representation of the underlying neural manifold and then performed community detection on the network to recover differences between behavioral states consistent with the “ground truth.”

A core method in TDA is “persistence homology,” which involves assessing the structure of a dataset at multiple scales by means of a “filtration” (e.g., Vietoris-Rips filtration). Without going into the mathematical details [again, see (Chazal and Michel, 2017) or (Perea, 2018) for specifics], the Vietoris-Rips filtration works by expanding balls around every point in the embedded point cloud, increasing the radius of each ball at a constant rate. When two balls intersect, an edge is drawn between the core points, creating a graph where geometrically close points are connected, while distant points remain disconnected. As the balls expand to a maximum radius (given by max(*D*(*i, j*))/2, where *D*(*i, j*) corresponds to some distance measure between two points *i* and *j*), the resulting network evolves, increasing in density as densely connected components and voids appear and disappear. The relationship with recurrence networks is obvious: every value of radius *r* in the Vietois-Rips filtration constructs a recurrence network with ϵ = 2*r*. In this way, persistence homology can be thought of as a generalization of the idea of a recurrence network that frees the user from having to select ϵ as a static free parameter: all possible values are searched instead. In addition to the insights gained from analyzing the whole filtration (which can be done with structures like bar-codes, persistence diagrams, Betti-curves, etc.) it is possible to “sample” key moments in the filtration, such as the moment the underlying network becomes connected, or the moment where variance of the degree-distribution is maximized, by treating the network at that moment as an RN and doing a more thorough analysis of the graph structure.

### 2.3. Applications of Recurrence Networks in Neuroscience

Recurrence-based analyses of EEG signals is a developing area of research in neuroscience, using both network and classical approaches. Recurrence networks have been found to help distinguish between healthy and epileptic brain activity (Subramaniyam and Hyttinen, 2013; Subramaniyam et al., 2015; Ngamga et al., 2016; Gao et al., 2017). Using TDA-based analyses of recurrence networks, Varley et al. (2021b) found distinct differences between awake macaques and those that had been anaesthetized with propofol or ketamine, such as a collapse of higher-order structure and a slowing of the “velocity” of the system in the embedded point clouds following propofol administration. A non-network-based techniques such as recurrence quantification analysis has been found to track changes to brain dynamics following anaesthesia (Li et al., 2007; Becker et al., 2010) or sleep (Song et al., 2004; Rolink et al., 2015), as has a closely-related measure, Poincare analysis (Hayashi et al., 2015).

The notion that, given a sufficiently large dataset, an RN can trace out a plausible “map” of the state-space of the system, complete with a “topography” of relatively higher or lower probabilities, allows for a detailed characterization of the emergent dynamics playing out in the brain complementary to static functional connectivity or cluster-based dynamic connectivity models. Returning to the notion of community detection in an RN, a dense community corresponds to a general pattern of activations across the cortex that the brain repeatedly returns to as it evolves through time. How “fuzzy” a pattern is (that is to say, how different two instantiations can be before we stop acknowledging them as “the same”) depends on the value of ϵ, but assuming a sufficiently dense community, it would be possible to take the average of the spatial distributions of activities across all nodes in the community and extract neurological information from that pattern.

RNs could be applied to a number of questions in computational and cognitive neuroscience. In the context of whole-brain analyses, the “map” provided by the RN can allow the comparison of different sizes, or “geographies” of neural state-space landscapes. Differences in the state-transition structure have been hypothesized to reflect a number of different mental states (Carhart-Harris and Friston, 2019). In the context of local circuits, attractor analysis can provide insights into the particular computations a network is performing (Breakspear, 2001).

## 3. Visibility Networks

Visibility networks (VNs) are arguably the most well-used method for constructing a network from a time series in neuroscience. In contrast to OPNs and RNs, the VN algorithm (described below) has no free parameters that need to be optimized, which contrasts favorably with methods which require selecting embedding dimensions, temporal lags, distance thresholds, etc. Unlike OPNs and RNs, the VN does not involve any kind of spatial embedding process, operating on the raw time series directly to construct the associated network. First proposed by Lacasa et al. (2008), the VN has been well-explored both analytically and experimentally, resulting in a rich literature of theory and applications (for review, see Zou et al., 2019, Section 4).

### 3.1. Constructing a Visibility Network

Like an OPN, a visibility network is typically constructed from a univariate time series, although multivariate extensions do exist, such a multi-layer, or multiplex network frameworks (Lacasa et al., 2015). In a visibility graph, each moment in the time series maps to a node in the network, and an edge exists between the nodes if they satisfy a “mutual visibility” condition.

Given a time series:


$$\begin{array}{l} {\text{X}}_{t}={\left(x_{1},x_{2},...,x_{t}\right)}_{|x_{i}\in \mathbb{R}} \end{array}$$

“Mutual visibility” can be understood by imagining **X**_*t*_ as a landscape and two points *x*_*i*_ at time *t*_*i*_ and *x*_*j*_ at time *t*_*j*_ are “mutually visible” if a person standing on *x*_*i*_ has an unobstructed line of sight to the person standing on *x*_*j*_. Formally, two points are mutually visible if, all values of *x*_*k*_ between *t*_*i*_ and *t*_*j*_ satisfy:


$$\begin{array}{l} \frac{x_{i}-x_{k}}{t_{k}-t_{i}}>\frac{x_{i}-x_{j}}{t_{j}-t_{i}} \end{array}$$

The VN can be made directed by including temporal information (i.e., edges can only point from past to future and not vice versa), although this is not necessarily standard practice. Constructing a VN, particularly for a long time series can be computationally expensive, as it requires testing the visibility condition a large number of times for every possible pair, and so an alternative, the “horizontal visibility network” (HGN), where a point is connected to the first moment in the future that has the same instantaneous amplitude. Formally, *t*_*i*_ and *t*_*j*_ are connected if, for all values *x*_*k*_ between them:


$$\begin{array}{l} x_{k}<\min\left(\left\{x_{i},x_{j}\right\}\right) \end{array}$$

**Figure 3:**
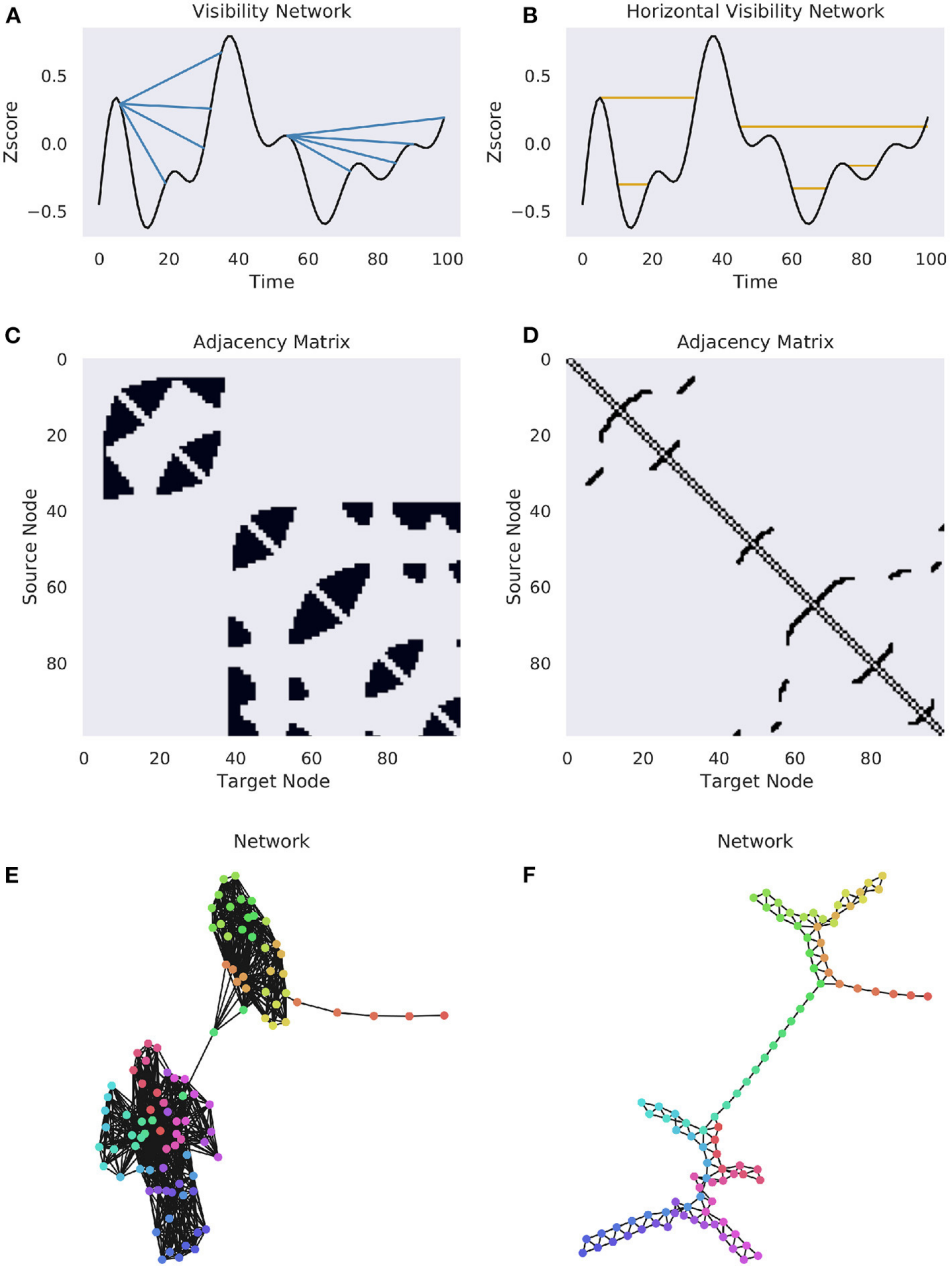
Complete and horizonal visibility networks. A comparison of the VN and HVN networks for the same Human Connectome Project BOLD data (Van Essen et al., 2013). A single time series was selected from a subject at random for demonstration purposes. **(A)** A cartoon of the visibility graph algorithm. For each origin point, an edge is drawn between it and all the points that it can “see”, illustrated by the multiple blue lines. **(B)** A cartoon of the horizontal visibility graph, where an edge is only drawn between an origin point at the next point at the same height. **(C,D)** The binary adjacency matrices for the above univariate time series. **(E,F)** The networks for the associated binary adjacency matrices. The colors correspond to the flow of time, as in Figure 1.

It is obvious that the HGV represents a subgraph of the natural VN, as all links in the HVN must appear in the natural VN. For a visualization of both methods of constructing a network, see Figure 3. The result of the VN algorithm is a network that will always be connected, un-weighted, undirected, and invariant under affine transformations of the original time series (Ahmadi et al., 2018).

### 3.2. Analyzing a Visibility Network

As with the OPN and RN, a significant benefit of the VN is that it can enable analyses of time series that can be difficult to do on continuous data. For example, the entropy of the degree distribution of an (H)GV has been used to approximate the entropy of a continuous time series (Gonçalves et al., 2016; Luque et al., 2016). This has certain benefits over more standard techniques of point-processing or discrete binning as the degree of a node in a VN encodes information about an excursion relative to the other points around it, as opposed to assuming that every moment is independently sampled from some underlying distribution. Similarly, this method of entropy estimation is not constrained by assumptions of Gaussianity in the original time series. This has enabled the development of information-theoretic analyses of continuous signals, such as the mutual information, or transfer entropy (Yu et al., 2017), which are ordinarily difficult to operationalize in the context of a continuous signal. Exactly how these measures compare to “standard” non-parametric estimators such as the KSG mutual information estimator (Kraskov et al., 2004) remains to be seen, although all Kraskov-based estimators require constructing nearest-neighbor networks, which make them prohibitive for long time series in a way that the VN is not. This may make them a less computationally intensive non-parametric estimator.

The degree of individual nodes is a core feature of VN analysis. Given a time series and its associated VN, it is possible to construct a symbolic time series where every *x*_*t*_ at time *t* is replaced with the degree of the associated node in the network *d*_*t*_. This time series is discrete and amenable to information-theoretic analysis, while still encoding temporal information from the original series. Furthermore, the degree distribution of the VN provides considerable insight into the dynamical regime of the original time series (Lacasa et al., 2008): fractal time series (i.e., Brownian motion) produce VNs with a scale-free degree distribution, while white noise produce networks with an exponential-random degree distribution. There are well-known, analytic solutions to the problem of constructing a visibility graph from random, unstructured data. For example (Luque et al., 2009) showed that, for a HVN, the degree distribution of the network follows an exponential form with a known value, regardless of the generating distribution of the original random data. Others have used the degree distribution of HVNs to discriminate between truly stochastic and deterministic, but chaotic, dynamics in temporal data (Ravetti et al., 2014). To the best of our knowledge, these insights have not yet been deployed on empirical data, but suggest that VNs may be useful for extracting stochastic (or noisy) components of data from deterministic (causal) ones.

Other standard network science measures such as community detection or density still want for interpretation to an extent.

### 3.3. Applications in Neuroscience

As previously mentioned, of the three methods of constructing a network from a time series presented here, visibility networks are the most well-explored, having been applied to multiple neural recording modalities. A particular focus has been the application of visibility graphs to assessing epileptiform activity in EEG data (Bhaduri and Ghosh, 2015; Gao et al., 2016; Liu et al., 2016; Supriya et al., 2016a,b; Wang et al., 2017; Zhang et al., 2018), although researchers have also used them to assess the differences between healthy and alcoholic volunteers (Zhu et al., 2014b), as well as the effects of stress (Ji et al., 2016), fatigue (Cai et al., 2018), sleep stages (Zhu et al., 2014a), autism spectrum (Ahmadlou et al., 2012), and Alzheimer's disease (Ahmadlou et al., 2010; Wang et al., 2016). Furthermore, natural VNs and HVNs have been used to explore the temporal irreversibility of EEG activity (Donges et al., 2013), which is an expanding area of interest based on the relationship between irreversibility, statistical complexity, and thermodynamic entropy (Lynn et al., 2021). One recent study took a multilayer VN approach to analyse fMRI data (Sannino et al., 2017) and showed that the multilayer VN enabled a novel, nonlinear analysis of multivariate interactions between brain regions and was sensitive to differences between control and disease states. VNs have also been applied to calcium imaging data as well (Zhu et al., 2018) for dynamic state detection in neuronal activity.

While VNs are, themselves quite amenable to assessing the dynamics of individual time series, they are also effective for inferring functional connectivity networks between time series (Sannino et al., 2017; Yu et al., 2017; Ahmadi et al., 2018). Different methods have been described, such as producing a multiplex network, effective connectivity networks, or synchronization-based measures, which leverage the network structure of the VN to assess the information contained in pairs of time series. A significant benefit of this method is that it allows for simultaneous analysis of individual time series, as well as higher order connectivity patterns using the same general framework: for example, a VN could be used to explore the relationship between the individual Hurst exponents of a pair of time series and their associated connectivity. This is a non-trivial problem, which can be avoided by deriving both the signal analysis and connectivity analysis from the same underlying VNs. As evidence accumulates that dynamics, in addition to connectivity are essential for complex cognition (Ezaki et al., 2020; Varley et al., 2020, 2021a,b) tools to simultaneously analyse dynamics and connectivity are likely to play a significant role in the future of computational neuroscience.

## 4. Ordinal Partition Networks

An ordinal partition network (OPN) provides a natural method of reconstructing a state transition network from a one dimensional time series produced by a system *X*_*t*_, such as an EEG signal, or LFP. In a state transition network, each node corresponds to one of a finite number of configurations that the system under study can adopt, and directed, weighted edges give the probability that the system will transition into a particular state, given the state it is currently occupying. Formally, given a graph *G* = (*V, E*):


(1) $$\begin{array}{r} E_{ij}=P\left(X_{t+1}=j|P\left(X_{t}=i\right)\right) \end{array}$$

State transition networks leverage the considerable work that has been done on finite-state machine analysis (Cover and Thomas, 2012) and are closely related to the idea of an ϵ-machine (Crutchfield, 1994, 2012), a provably optimal symbolic model of a dynamical process.

**Figure 4:**
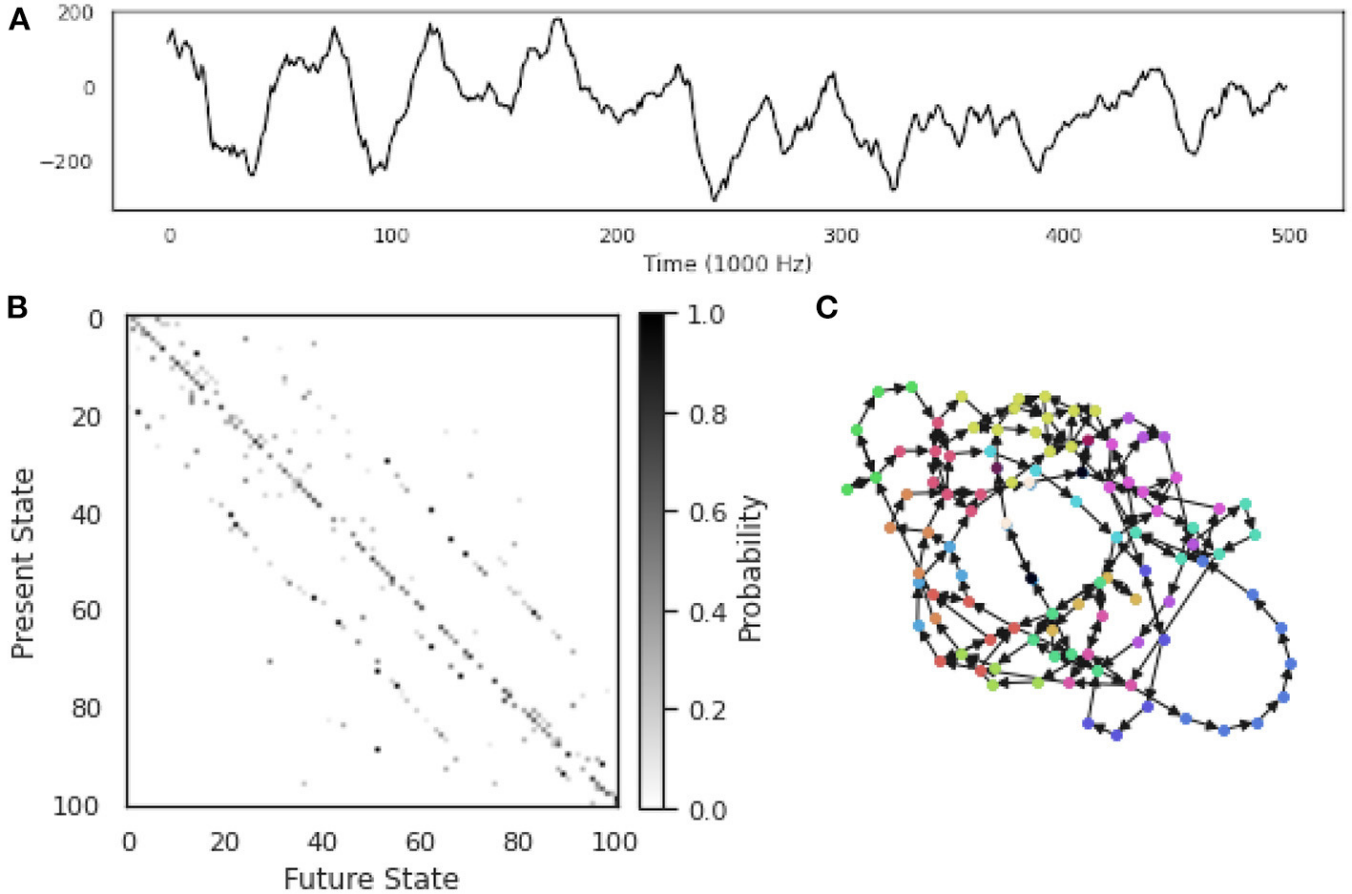
Constructing an Ordinal Partition Network from a single time series. An example of an OPN constructed from a single ECoG time series taken from the Neurotycho database (Nagasaka et al., 2011). **(A)** The time series itself: an electrophysiological time series recorded using invasive intra-cortical arrays. **(B)** The transition probability matrix for the OPN. **(C)** The OPN itself, nodes colored by community assignment determined using the Informap algorithm (Rosvall and Bergstrom, 2008; Rosvall et al., 2009). Note the mixture of long, path-like cycles corresponding to rare excursions through state-space, as well as denser regions with a high degree of interconnectivity.

Described in detail below, OPNs are based on permutation embeddings (Bandt and Pompe, 2002; Riedl et al., 2013), which map a continuous, real valued time series to a series of discrete symbols (typically strings of numbers) taken from a finite-sized dictionary. This effectively coarse-grains the time series, and from this symbolic series, transition probabilities can be easily constructed by counting the number of times two symbols appear in sequence. The OPN can be thought of as a symbolic approximation of the attractor driving the original, continuous dynamics (Small, 2013; McCullough et al., 2015), and frequently makes use of classical Taken's phase-space embedding procedures (Takens, 1981) to reconstruct the attractor model. The utility of such a state transition network is that, by coarse graining the continuous signal and forming a finite-state machine, the door is opened to a large number of information-theoretic and computational analyses that are not naturally applicable to continuous data (for a visual summary of OPNs, see Figure 4).

Currently, OPNs are almost exclusively used on univariate time series, although mulivariate generalizations have been proposed. Zhang et al. (2017) explored a multidimensional generalization based on the first derivative of the time series: permutations are assigned based on the direction, and magnitude, of the change in value at each timestep, for each time series, although this method has not yet been well-explored. Related alternatives, developed to assess the dependency between time series, are the joint and cross OPNs (Guo et al., 2018), which have been successfully used to characterize complex systems, such as interacting climatic systems (Wu et al., 2020). An issue that all multivariate OPN construction algorithms run into is the explosion of the number of possible joint states observable from high-dimensional time series. As the number of dimensions grows, the amount of raw data required to infer reliable estimates for the entire joint probability space explodes, which restricts the space of possible applications. For example, without dimensionality reduction, there is likely no practical way to construct a multidimensional OPN from a 128-channel EEG dataset, regardless of the specific algorithm chosen.

### 4.1. Constructing an OPN

Permutation embeddings were first introduced by Bandt and Pompe as a natural method of discretizing a continuous, real-valued time series. As previously discussed, many information theoretic analysis of interest to scientists working with time series data do not readily generalize to continuous data, as they require a finite number of discrete, non-overlapping states on which probability distributions can be defined (Cover and Thomas, 2012). The permutation embedding defines the “state” of a time series at a given time *t* as a temporally-extended pattern of fluctuations which begins at *t* and ends at some later time. Given a time series:


$$\begin{array}{l} {\text{X}}_{t}={\left(x_{1},x_{2},...,x_{t}\right)}_{|x_{i}\in \mathbb{R}} \end{array}$$

the first step is a standard time-delay embedding with embedding dimension *d* and time-lag τ such that, for all *i*∈*t*:


$$\begin{array}{l} V_{i}=\left(x_{i},x_{i+\tau },x_{i+2\tau },...,x_{i+\left(d-1\right)\tau }\right) \end{array}$$

The result is, for all *x*_*i*_ in **X**_*t*_, we have created an associated vector embedded in ℝ^*d*^. To discretize these vectors, we assign an ordinal rank (in ascending order) to each element of *V*_*i*_:


$$\begin{array}{l} S_{i}=\pi_{1}\pi_{2}...\pi_{d},\;\pi_{i}\in \left\{1,2,..d\right\},\;,\pi_{i}\neq \pi_{j} \end{array}$$

This discretizing operation is typically denoted as ϕ(*V*_*i*_). As an example, consider the time series:


$$\begin{array}{l} {\text{X}}_{t}=\left(0.1,0.3,1.2,0.2,0.8,1.7,1.3,0.4,0.2\right) \end{array}$$

Embedding with *d* = 3 and τ = 2 returns:


$$\begin{array}{l} V_{1}=\left(0.1,1.2,0.8\right)\\ V_{2}=\left(0.3,0.2,1.7\right)\\ V_{3}=\left(1.2,0.8,1.3\right)\\ V_{4}=\left(0.2,1.7,0.4\right)\\ V_{5}=\left(0.8,1.3,0.2\right) \end{array}$$

Applying the ϕ operator:


$$\begin{array}{l} S_{1}=\phi \left(V_{1}\right)=132\\ S_{2}=\phi \left(V_{2}\right)=213\\ S_{3}=\phi \left(V_{3}\right)=213\\ S_{4}=\phi \left(V_{4}\right)=132\\ S_{5}=\phi \left(V_{5}\right)=231 \end{array}$$

The sequence **S**_*t*_ = (132, 213, 213, 132, 231) corresponds to the permutation embedding of **X**_*t*_. Note that we do not include values *x*_*i*_ where *i*>τ × *d*, as these would produce embedded vectors of less than *d*-dimensions. The ϕ operator makes the OPN remarkably robust to outliers in the data: since the absolute value of a sample is irrelevant (only the relative extremity is relevant), high-amplitude outliers are “damped” (contrast this with VNs where outliers can take central roles in the network).

To construct the OPN *G*_*O*_, each *S*_*i*_ maps to a node in *G*_*O*_, and the weighted, directed edge *e*_*ij*_ between nodes *i* and *j* is given by the probability that *S*_*i*_ is followed by *S*_*j*_. Continuing with our example, the node corresponding to 132 has two out-going edges, each with weight 0.5: one going to 213 and another going to 231. The weights of the out-going edges define a probability distribution over the possible futures of a random walker standing on node *i*.

#### 4.1.1. Selecting *d* and τ

**Figure 5:**
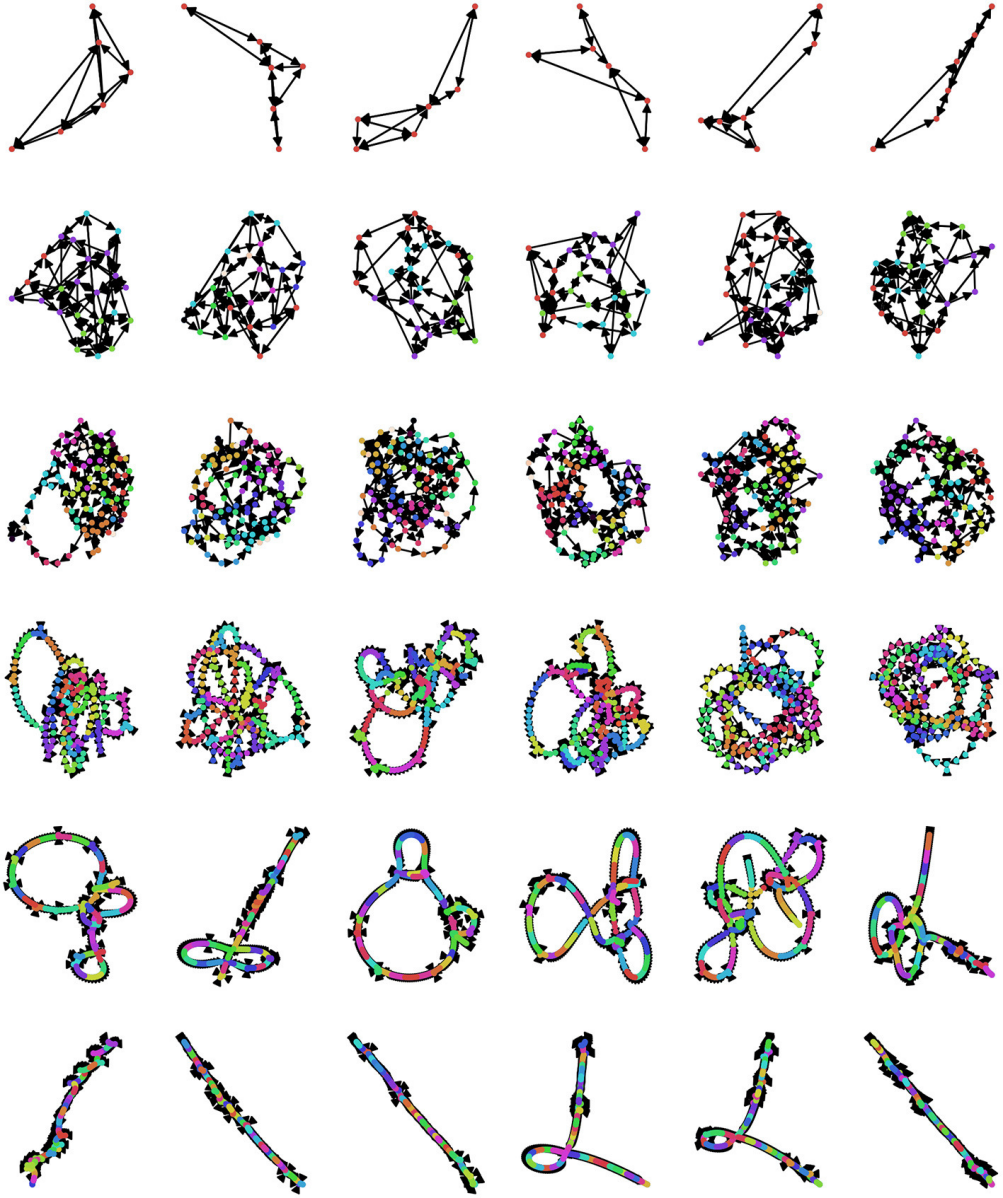
Exploring the space of possible ordinal partition networks. Possible OPNs for a single time series with lags ranging from 12 to 24 (rows, in increments of 2) and dimensions ranging from 3 to 8 (columns). Nodes are colored according to community assignment determined using the Infomap algorithm (Rosvall and Bergstrom, 2008; Rosvall et al., 2009). Note that, as the embedding dimension gets large, the networks become increasingly path-like, as every embedded vector gets it's own unique ordinal partition vector, creating an illusion of determinism. The data is the same Neurotycho time series as what was used above in Figure 4.

Currently, there is no universally agreed-upon method for selecting the embedding dimension *d* and lag τ (Zou et al., 2019), although several different criteria have been suggested. An optimal value of *d* should meet several standards: if it is too small, then the associated OPN will quickly become “saturated,” as all possible ordinal partitions will appear, obscuring more complex dynamics (this can be thought of as a kind of excessively lossy compression of the time series). In contrast if *d* is too large, then every moment in the resulting ordinal partition embedding will be unique and so the time series is simply recreated as a path graph. For visualization, see Figure 5, where, for small embedding dimensions (top row), all possible permutations are realized, while the network gets progressively more path-like as embedding dimension increases. One commonly-used method for selecting *d* is the False Nearest Neighbors technique (Kennel et al., 1992; Ruan et al., 2019; Wu et al., 2020), which finds the minimum number of dimensions that preserves local relationships between points. Alternately, given some pre-defined lag τ, the optimal value of *d* can be defined as the first peak of the permutation entropy over a range of values of *d* (Riedl et al., 2013; Myers et al., 2019). Another method that takes advantage of the network structure of the OPN is to select a value of *d* that, given some pre-selected lag τ, maximizes the variance in the degree distribution of the resulting network, which was found to create networks that most closely captured the structure of continuous attracts such as the Rossler attractor (McCullough et al., 2015). This measure has appeal as a natural solution to the original problem of constructing an optimal network on a continuum between a complete graph and a path graph: the network with the highest-variance degree-distribution may be thought of as the graph with the “richest” internal structure. In a context where the researcher wants to compare two OPNs from distinct time series, it may be necessary to ensure that the value of *d* for both time series is the same, thus forcing both OPNs to be constructed from a common alphabet (in the “same language,” as it where). In this case, we recommend selecting the smallest value of *d* for which both networks are unsaturated.

When selecting the value of the embedding lag τ, it is common practice to define it as the first zero of the autocorrelation function (McCullough et al., 2015; Zou et al., 2019). While this is by far the most commonly-used heuristic, it comes with the significant limitation that it is only sensitive to linear relationships within the time series under question. McCullough et al. (2015) suggests using the auto-mutual information (Fraser and Swinney, 1986) as a generalization sensitive to non-linearities in the dataset. As one of the key benefits of information-theoretic analyses, such as permutation embedding and OPN construction, is that they are model-agnostic, using an autocorrelation measure that throws out non-linearities in the data seems to defeat the purpose somewhat. A significant difficulty of auto-mutual information measures, however, is that they are often not naturally applicable to continuous, real-valued time series, so some binning procedure is likely to be required (for a discussion of mutual information estimators for continuous data, see Papana and Kugiumtzis, 2009). Recently, much more involved algorithms for automatic optimization of *d* and τ based on frequency-domain analysis have been proposed as well (Myers and Khasawneh, 2020), and debate on this question is likely to be on-going for the foreseeable future. One possibility that has not been explored is selecting parameters that produce surrogate data that best preserves some feature of interest in the original data: (McCullough et al., 2017) provides an algorithm for how a constrained random walk on an OPN can be used to generate synthetic time series that preserve the ordinal partition transition dynamics of the original time series and so *d* and τ might be selected as the values that create OPNs than produce synthetic data that has (for example) the frequency band profile most like the original data.

There has been little work assessing the effects of noise contamination on OPNs constructed from empirical time series. However, it is known that the OPN associated with unstructured, white noise has a surprising degree of non-trivial structure (Pessa and Ribeiro, 2019) that does not correspond to a either simple Erdos-Renyi graph (the characteristic random null model in network science) or a complete graph (since certain transitions are impossible). Furthermore, Pessa and Ribeiro (2019) found that there was a characteristic pattern of changes that emerged as purely periodic OPNs were increasingly contaminated with noise: the number of unique nodes in the networks rapidly increased to the maximum, but the overall transition structure remained the same (i.e., those nodes existed, but had low transition probabilities). It was only after the series was strongly contaminated with noise that the transition structure of the network began to take on the characteristics of a truly random OPN.

### 4.2. Analyzing an OPN

The OPN encodes a number of relevant features of the original dynamics in its topology, and even simple graph metrics can provide a rich picture of the dynamics present in the original time series. The simplest measure is the number of nodes in the network (corresponding to the number of unique ordinal partitions in the original embedding). This provides an estimate of the “richness” of the original time series, and the size of the repertoire of states available to the generating process. The entropy, or variance, of the number of times each node is visited corresponds to Bandt and Pompe's original measure of permutation entropy (Bandt and Pompe, 2002). Work with modeling well-understood dynamical systems (e.g., Rossler or Lorenz systems) with OPNs has found that the distribution of out-going edges (mean out-degree or variance in out-degree distributions) increases dramatically near the onset of deterministic chaos (McCullough et al., 2015). As the evolution of chaotic systems is associated with a high degree of long term unpredictability, this makes intuitive sense. A related set of measures, the determinism and degeneracy of the process can be related to the entropy of the out-degrees as well (Klein and Hoel, 2020). Determinism gives a measure of how reliably the future can be predicted based on the past, while degeneracy, in contrast is a measure of how much information about the past is lost when distinct past states flow into the same present state (for review, see Hoel et al., 2013; Klein and Hoel, 2020). As with other out-degree-based measures, determinism and degeneracy appear to be sensitive to the dynamical regime the system is in Varley (2020). Analysis of a diode resonator circuit found that, near the critical boundary between periodicity and chaos (the bifurcation cascade), the network diameter diverged (McCullough et al., 2015), and in a Rossler system, the presence of higher order community structure appeared to collapse around the critical point as well (Varley, 2020).

Being weighted, directed graphs, OPNs are not assumed to exhibit transitional symmetry (i.e., *p*_*ij*_≠*p*_*ji*_). The degree of asymmetry can be quantified in several ways, such as a ratio between |*p*_*ij*_−*p*_*ji*_|/(*p*_*ij*_+*p*_*ji*_) as in (Masoller et al., 2015), or as a measure of entropy-production: $\underset{i,j}{\sum }p_{ij}\log\left(p_{ij}/p_{ji}\right)$, as in Lynn et al. (2021). In systems near a thermodynamic equilibrium, the associated OPN should generally be symmetrical, while systems far from equilibrium should have obvious, non-random flows through state-space (Lynn et al., 2021). The association between irreversibility, complex statistical state transition dynamics, and thermodynamic processes is an emerging field of research (Roldan and Parrondo, 2010, 2012; Masoller et al., 2015) and OPNs provide a natural avenue to assess these questions in empirical time series data.

#### 4.2.1. Community Detection

As far as we know, there has not yet been an explicit treatment of the question of community detection in OPNs, or state transition networks more broadly, although we consider this a promising area of future study. Standard definitions of a community in a network typically hinge on a greater degree of within-community edges than between-community edges (Barabási and Pósfai, 2016), and in the context of an OPN (or any state transition graph) a community can be understood as a subset of states that the system gets transiently “stuck” in as it evolves through time. This logic is already the basis of multiple community detection algorithms in network science, such as the Walktrap algorithm (Pons and Latapy, 2005) and the Infomap algorithm (Rosvall and Bergstrom, 2008; Rosvall et al., 2009). For an OPN, following community detection with an algorithm like Infomap, the original network can be “renormalized” so that each community in the “micro-scale” network maps to a node in the “macro-scale” network (Klein and Hoel, 2020), where causally similar micro-states are aggregated. There are clear parallels here to the notion of “metastability” (Cocchi et al., 2017), where a system is described as metastable if its behavior is dominated by winnerless competition between attracting “macro-states” (in this case, communities in the network that the walker gets transiently trapped in).

### 4.3. Applications in Neuroscience

Being comparatively novel, the use of OPNs in neuroscience has been limited. Ref. Mccullough (2019) found that OPNs can differentiate between EEG data from healthy and epileptiform activity in a machine learning task. Myers et al. (2019) included EEG data in their analysis of persistent homology of OPNs as a proof-of-concept for physiological signals although there was no neuroscientific question being investigated in that paper. The most detailed application of OPNs to neuroscientific questions was done in Varley et al. (2021b), where OPN-based measures were found to be highly sensitive for discriminating between healthy, waking brain states and those states induced by anaesthetics ketamine and propofol. A key feature of the anesthesia analysis is that the OPN naturally provides a battery of measures that can be used to characterize brain dynamics (number of unique micro-states, determinism/degeneracy, metastability, etc.). This contrasts with much of the previous work that has been done which focuses on single point measures of brain complexity such as Lempel-Ziv compressibility (Schartner et al., 2015, 2017) or integrated information (Toker and Sommer, 2019).

The metastability analysis described above may be of particular interest to neuroscientists, as metastability has been proposed as a key feature of brain dynamics essential for cognitive flexibility and learning (Tognoli and Kelso, 2014; Cocchi et al., 2017). Consequently, a potentially fruitful avenue of research might be using OPNs to characterize individual differences at the level of the dynamics of individual brain regions. Researchers have found that analysis of dynamics from single brain regions tracks differences such as ageing, development, and differences such as autism spectrum disorder (Grady and Garrett, 2014; Nomi et al., 2017; Easson and McIntosh, 2019). Given appropriate data, an OPN provides a rich feature-space that can be explored to assess the similarities and differences between dynamical processes [previously referred to as a “dynamical morphospace.” (Varley et al., 2021b)]. This could be used to provide a detailed exploration of state- and individual-differences between subjects. For example, are individuals who score more highly on tasks related to creativity have more flexible state-transition dynamics? Or are the deficits associated with Alzheimer's associated with a contraction of the repetoire of available states? Finally, since the OPN can be constructed for each region individually (out of the corresponding, univariate time series), regional differences and their differential contributions can be assessed as well.

While OPNs are typically restricted to univariate time series, the interaction of multiple time series by analyzing the coupling between their associated OPNs may provide a path forward. A suite of measures based on permutation embeddings as proposed by Ruan et al. (2019) to assess information flow between coupled time series, which could be applied to the construction of functional or effective connectivity networks across the whole brain. Furthermore, measures of overall network structural similarity, such as Bagrow and Bollt (2019) can provide a measure of the “dynamical similarity” between two processes. How these functional connectivity networks may differ from more standard measures, such as correlation-based associations remains to be explored.

Finally, an OPN can be used to generate surrogate time series data (McCullough et al., 2017) which preserve the information dynamics of the original. Null-models are of crucial importance in modern, computational neuroscience, ensuring that an observed effect is “real” (Moore, 1999; Rubinov, 2016). While much work has been done developing measures for generating null time series that preserve measures such as autocorrelation or other properties (Lancaster et al., 2018), data generated by a random walk on the OPN preserves information dynamics in a way that, to the best of our knowledge, has not been done before. An important future line of research will be determining what features of a continuous time series (frequency spectrum, autocorrelation, etc.) are also preserved using this method.

## 5. Software Implementations

At the time of this writing, there are no universal packages that will construct and analyse OPNs, RNs and VNs from provided time series data, although individual packages do exist. For those interested in recurrence networks and visibility graphs, the Python package pyunicorn (Donges et al., 2015) provides fast implementations of both. Written in Python, C/C++, and Fortran, pyunicorn can handle long time series and large networks. Although primarily aimed at researchers working with climatological data, it should be useful for any neuroscientists interested in recurrence networks and visibility graphs.

As far as we can tell, there are no publicly available implementations for constructing OPNs from time series data. To that end, we are providing one: OPyN is a Python-based package for rapid construction and analysis of ordinal partition networks and permutation embeddings. Built in Cython (Behnel et al., 2011) and Python-iGraph (Csardi and Nepusz, 2006), OPyN can quickly embed long time series, construct large networks, and help identify optimal values of the embedding dimension *d* and the time-lag τ. Code is available on Github. OPyN can also be incorporated into C code, thanks to cross-comparability provided by Cython.

Here we will briefly review the functions native to OPyN and illustrate how they might be used to analyse neural data. Given a 1-dimensional time series (BOLD, electrophysiological, LFP, etc.), OPyN provides two functions for selecting the optimal embedding procedures: optimal_lag() will return the delay corresponding to the first zero of the autocorrelation function (McCullough et al., 2015; Zou et al., 2019) and optimal_dim() will estimate the optimal embedding dimension as the dimension that maximizes the variance in the OPN's degree distribution, given some lag (McCullough et al., 2015). Given these two parameters, the user can either construct the entire OPN using the OPN() function [which returns a directed, weighted python-igraph (Csardi and Nepusz, 2006) object], or create the permutation-embedded time series using the permutation_embedding() function. If the user wishes to use the OPN as a generative model to produce surrogate time series that preserve the dynamics of the system, the function constrained_walk implements the algorithm details in McCullough et al. (2017). While the package was written by neuroscientists, the OPN framework is very general and can be applied to a number of fields, such as climate modeling (Wu et al., 2020) and researchers outside of neuroscience should feel at liberty to be creative when using this package.

## 6. Conclusions

In this work, we provide an introduction to three methods to analyse nonlinear physiological signals by constructing network models that map temporal dynamics to topological structures. The first method is the recurrence network (RN) (Donner et al., 2010) (Section 2). The RN is constructed by treating a classical recurrence plot as the adjacency matrix for an undirected network, where two nodes are connected if they are closer to each other than some minimum metric distance (ϵ). Consequently, frequently visited regions of phase-space are characterized by the emergence of densely connected subgraphs, while sparsely visited, or “forbidden” configurations appear as voids. Unlike the OPN and the visibility graph, the RN is the only one of the three that has a natural embedding in metric space, which can be visualized using topology-preserving techniques such as multidimensional scaling (Borg and Groenen, 2005) or UMAP (McInnes et al., 2018). Of the three algorithms described here, RNs are the most suited for work with very high-dimensional data, and are also more suited for shorter recordings (as is common in fMRI studies), although short, artifact-free slices of M/EEG or LFP data could also work (as was done in Varley et al., 2021b). RNs are also related to techniques from Recurrence Quantification Analysis (RQA) (Marwan et al., 2007) and topological data analysis (TDA) (Chazal and Michel, 2017). Computationally, the RN is the most intensive, requiring computing the distance between every point (represented in memory as a two-dimensional array of floating-point values) in the time series and runs in approximately *O*(*n*^2^) time. If the network is then thresholded, sparse matrix representations can cut down on computational overhead, but the initial inference is exhaustive.

The second method introduced in this paper is the visibility network (VN) (Lacasa et al., 2008) (Section 3). In a VN, an edge exists between two nodes if they satisfy a mutual visibility condition (i.e., if two people standing on two differing parts of the time series can see each other). The VN can be naturally applied to both univariate time series data (as was done in the classic VN and HVN), or expanded to multivariate time series using a multi-layer network approach (as was done in Sannino et al., 2017 using fMRI data). This makes VNs amenable to both low-temporal resolution data such as fMRI, as well as high-frequency data such as M/EEG data.

The VN naturally encodes extreme events as hubs (since most points on the network can see a large excursion from baseline), and it is well-documented that different dynamical regimes translate into distinct VN structures: for example, fractal time series map to scale-free networks, while random networks show an exponential-random degree distribution (Lacasa et al., 2008). This tendency for the VN to assign a central location in the network to extreme events brings with it the added caveat that an outlier artefact from recording could interfere with the analysis of the network in a way that is not the case in a symbolic network like an OPN. Consequently, care should be taken when preparing time series data for analysis. Computationally, inferring a VN is similar to an RN, although the restriction on “looking backwards” means that the runtime complexity is decreased by a factor of two *O*(*k*^2^/2). The resulting networks are typically sparse, allowing for efficient representation in memory.

The final method is that of constructing ordinal partition networks (OPNs) from univariate time series (Section 4). The OPN, based on the notion of a discrete, permutation embedding (Bandt and Pompe, 2002), maps a continuous signal to a finite set of “state words” and then constructs a state transition network based on the probability of transitioning to a given state conditioned on the current state (McCullough et al., 2015). The OPN encodes a large number of dynamical properties in its structure, such as the relative size of the repertoire of available states (number of nodes), the determinism and degeneracy of the original signal (from the entropy of out-degrees), and the presence/absence of higher-order “emergent” dynamics (community structure) (Klein and Hoel, 2020; Varley, 2020). Furthermore, the OPN is arguably the most amenable to information-theoretic analysis, based on the large literature applying such analyses to finite-state machines (Cover and Thomas, 2012). Unlike RNs and VNs, however, the OPN is not currently well-equipped to handle multivariate time series of more than two or three dimensions, instead being highly optimized for univariate analysis. Furthermore, it requires the most data to effectively estimate the full joint transition probability matrix. This makes it optimal for electrophysiological recordings with high temporal density (as was done in Varley et al., 2021b).

Of the three algorithms, the OPN is the most computationally efficient, requiring the permutation embedding for every point in the series, and then the construction of the state-transition network, which can be done with a runtime complexity of approximately *O*(*k*) (given that the embedding dimension is rarely greater than five, the runtime on the sorting algorithm is negligible). Like the VN, the resulting OPN network is also typically sparse, allowing for efficient representation in memory.

A key question in modern complex systems approaches to neuroscience is understanding the relationship between on-going brain dynamics, cognition, and behavior (Shine et al., 2018, 2019a). This is naturally a more involved question than assessing what elements of the nervous system interact and requires the ability to describe time-resolved activity (Lizier, 2014; Esfahlani et al., 2020; Sporns et al., 2021), and the flow of the system over an “intrinsic manifold” (Huys et al., 2014; Shine et al., 2018; McIntosh and Jirsa, 2019) that can describe how different dynamical regimes map onto distinct cognitive states (Cocchi et al., 2017), both in health and disease. Network analysis of time series is a natural set of tools to use to tackle these questions. Since every node maps to a particular moment in time, it is easy to do both time-resolved analysis (corresponding to understanding the local neighborhood around a given node) and characterize global patterns (corresponding to whole network structures). When using networks to analyse time series, the relationship between local and global network analysis naturally correspond to time-resolved and average temporal properties.

Network analysis of time series may prove useful in clinical practice, where considerable focus is on assessing differences between states such as health, anaesthesia (Varley et al., 2020, 2021b), sleep (Zhu et al., 2014a; Schartner et al., 2017; Chaudhuri et al., 2019), disorders of consciousness (Comolatti et al., 2019; Luppi et al., 2021), or neurobiological illnesses (Subramaniyam and Hyttinen, 2013; Subramaniyam et al., 2015; Ngamga et al., 2016; Gao et al., 2017). More subtle comparisons may also be possible, such as local or global differences between rest and task states (Shine et al., 2018, 2019a). As all of these methods can be deployed on univariate time series, it is also possible to do finer grain analysis at the level of the dynamics from individual brain regions (as in Zhu et al., 2014b).

There are some limitations inherent in network analysis of time series. One limitation is that many of these methods require sufficiently long time series to get a full picture of the dynamics. While this is not typically an issue for M/EEG methods, for fMRI analysis which often have BOLD signals with less than a few hundred samples, this can represent a significant hazard. The most obvious limitation, as mentioned in the introduction, is that these networks trade spatial resolution for temporal resolution, typically collapsing many brain regions into a single mathematical object (although this not universal, for example the RN can preserve information about the distribution of activities across the channels). An area of future research might be the development of higher-order OPNs and VNs that can handle multidimensional time series.

The field of network analysis of time series is still novel, and its application to neuroscience even moreso. This opens the door to a rich area to explore at the intersection of information-theoretic, dynamic, and network science-based approaches (for a discussion of the intersection of dynamical systems and computational approaches to neuroscience, see Mediano et al., 2021; Varley et al., 2021a). By enabling time-resolved inference of complex state-spaces, network analysis of time series allows researchers to leverage the considerable power of network science and graph theory to questions of neural activity in ways that were not previously enabled by “classical” network neuroscience, and we are optimistic that these new methods will provide a powerful, complementary branch of network neuroscience with which to explore brain structure and function.

## Author Contributions

TV conceptualized the project and wrote the initial manuscript. OS provided supervision and edits to the manuscript. All authors contributed to the article and approved the submitted version.

## Funding

TV was supported by NSF-NRT grant 1735095, Interdisciplinary Training in Complex Networks and Systems.

## Conflict of Interest

The authors declare that the research was conducted in the absence of any commercial or financial relationships that could be construed as a potential conflict of interest.

## Publisher's Note

All claims expressed in this article are solely those of the authors and do not necessarily represent those of their affiliated organizations, or those of the publisher, the editors and the reviewers. Any product that may be evaluated in this article, or claim that may be made by its manufacturer, is not guaranteed or endorsed by the publisher.
